# What are economic costs and when should they be used in health economic studies?

**DOI:** 10.1186/s12962-023-00436-w

**Published:** 2023-05-15

**Authors:** Hugo C. Turner, Frank G. Sandmann, Laura E. Downey, Stacey Orangi, Yot Teerawattananon, Anna Vassall, Mark Jit

**Affiliations:** 1 MRC Centre for Global Infectious Disease Analysis, School of Public Health, Imperial College London, London, UK; 2 Department of Infectious Disease Epidemiology, London School of Hygiene and Tropical Medicine, London, UK; 3 The George Institute for Global Health, School of Public Health, Imperial College London, London, UK; 4 The George Institute for Global Health, University of New South Wales, Sydney, Australia; 5 Health Economics Research Unit, KEMRI-Wellcome Trust Research Programme, Nairobi, Kenya; 6 Health Intervention and Technology Assessment Program (HITAP), Ministry of Public Health, Nonthaburi, Thailand; 7 Saw Swee Hock School of Public Health, National University of Singapore, Singapore, Singapore; 8 Department of Global Health and Development, London School of Hygiene and Tropical Medicine, 15-17 Tavistock Place, London, WC1H 9SH UK; 9 School of Public Health, University of Hong Kong, Hong Kong Special Administrative Region, China; 10 Present Address: European Centre for Disease Prevention and Control (ECDC), Stockholm, Sweden

**Keywords:** Economic costs, Opportunity costs, Decision making, Definition, Rationale, Health economics, Economic evaluations

## Abstract

Economic analyses of healthcare interventions are an important consideration in evidence-based policymaking. A key component of such analyses is the costs of interventions, for which most are familiar with using budgets and expenditures. However, economic theory states that the true value of a good/service is the value of the next best alternative forgone as a result of using the resource and therefore observed prices or charges do not necessarily reflect the true economic value of resources. To address this, economic costs are a fundamental concept within (health) economics. Crucially, they are intended to reflect the resources’ opportunity costs (the forgone opportunity to use those resources for another purpose) and they are based on the value of the resource's next-best alternative use that has been forgone. This is a broader conceptualization of a resource’s value than its financial cost and recognizes that resources can have a value that may not be fully captured by their market price and that by using a resource it makes it unavailable for productive use elsewhere. Importantly, economic costs are preferred over financial costs for any health economic analyses aimed at informing decisions regarding the optimum allocation of the limited/competing resources available for healthcare (such as health economic evaluations), and they are also important when considering the replicability and sustainability of healthcare interventions. However, despite this, economic costs and the reasons why they are used is an area that can be misunderstood by professionals without an economic background. In this paper, we outline to a broader audience the principles behind economic costs and when and why they should be used within health economic analyses. We highlight that the difference between financial and economic costs and what adjustments are needed within cost calculations will be influenced by the context of the study, the perspective, and the objective.

## Background

Setting healthcare policy requires consideration of not only the benefits of health interventions but also what resources it requires. Therefore, both benefits and costs need to be appropriately considered and captured to inform evidence-based decision making. However, within the field of public health, many are unaware of how the interventions should be appropriately costed, particularly within economic evaluations [1]. The use of health economic evidence to inform decision making and the allocation of healthcare resources is becoming more relevant, as ageing populations will increasingly drive greater resource needs across the world.

Fundamentally, the resources available for healthcare are limited, making it impossible to implement every possible health intervention. The scarcity of resources requires us to constantly make choices between alternatives in almost every situation, implicitly or explicitly. By pursuing one action, the potential benefit that could have been gained from the next-best alternative action is sacrificed—which is known as an opportunity cost. Some have argued that opportunity costs exist only in the “eye of the beholder” as the envisioned “alternatives” do not actually occur and cannot be measured by outsiders [2]. Irrespective of differences between economists, the key concept of opportunity costs is intended to capture the competing use of limited resources and the need to make choices.

Crucially, within healthcare there are inevitably trade-offs for the available resources, such as the time of healthcare staff, beds and equipment: using resources on a particular patient means there is a lost opportunity to use the same resources on another at the same point in time (for example in some countries there are patient waiting lists, where the demand for hospital inpatient care exceeds the available beds). More formally, the opportunity cost of making a particular choice is the value of the next-best alternative that is forgone. This is a fundamental concept within (health) economics and healthcare decision making [1], as summarised by Selma Mushkin as far back as 1958 [3]: “The health administrator has usually equated ‘health economics’ with ‘money questions in the field of health.’ But, money is not the central problem of health economics. Health economics is concerned with the optimum use of scarce economic resources for the care of the sick and the promotion of health, taking into account competing uses of these resources”.

Economic theory states that the true value of a good or service is its value of the next best alternative forgone as a result of using the resource i.e. its opportunity cost. This is distinct from the idea of financial costs (the financial expenditures that are actually paid for a good or service) which do not necessarily reflect the true economic value of a good or service. For example, there would not be a financial cost (i.e. salary) associated with the time a community health volunteer donates to an intervention. However, there will be an opportunity cost associated with the donated time, as the volunteers have had to forgo some other activity (such as paid work) in order to spend the time on the intervention [4]. It is standard practice within many types of health economic studies to use what is known as economic costs [1, 5, 6]; which reflect the full value of the resources utilized in providing an intervention rather than the amount paid for them. They are intended to reflect the forgone opportunity to use those resources for an alternative purpose (i.e. their opportunity cost) [7].

Health economics is increasingly applied in interdisciplinary contexts and the use of health technology assessment, including economic evaluations, is expanding globally. As a result, clinicians and public health professionals are being increasingly called upon to contribute and apply economic evaluation evidence. However, the current literature on economic costs is focused on an economist audience and those who have a prior understanding of economic terminology. Consequently, there is a risk that economic costs and the reasons why they are used may be misunderstood by public health professionals and clinicians using or contributing to the economic evidence base [1].

This paper aims to introduce and raise awareness of the difference between financial and economic costs, and outline when economic costs are needed within health economic analyses. It is targeted to an audience of public health professionals who are conducting or interpreting health economic studies without an economic background. As such, it aims to fill the gap in providing a comprehensive introduction to these concepts targeted at a broader audience of non-economists, acting as an entry point to the more technical guidance available. Crucially, although the principles raised apply to any setting, the examples provided are particularly relevant to a global health context [including low and middle-income country (LMIC) settings], where there is often more limited capacity in health economics [8]. Greater awareness of these concepts will aid in leading to improved consistency in future health economic studies and is important as when these costing concepts are not understood (such as the reasons why different results can be obtained through different methodological choices), it could ultimately lead to inefficient policy decisions.

### The cost of health interventions: financial costs versus economic costs

The costs of interventions are a vital consideration in policymaking within healthcare. There are three steps involved in estimating both financial and economic cost values: (1) identifying what resources are used within the study’s perspective, (2) measuring the amount of each resource used, and (3) placing a monetary (or non-monetary) value on each resource [4]. The fundamental difference between financial and economic costs surrounds the last step regarding the valuation of the resources. Importantly, for both financial and economic costs, which resources are included in the costing of an intervention depends on the adopted perspective for the analysis. This is the point of view adopted when deciding which types of costs and benefits are to be included (for example this could be the costs incurred by the individual patient, the healthcare provider or society as a whole) (outlined further in Box 1). The choice of perspective will be linked to what is the objective of the study is.

**Box 1:** The study’s perspective, timeframe and sunk costs

The perspective of a health economic study is the point of view adopted when deciding which types of costs and benefits are to be included [9]. Potential perspectives include the patient, a specific payer (such as a specific control programme), the healthcare provider, the healthcare sector, or the broader society as a whole (the societal perspective—where all relevant costs, regardless of who they are incurred by are included)	
In the context of economic costs, the study’s perspective influences what resources are included and from whose point of view the opportunity costs are quantified. For example, the opportunity cost of the patients’ time to access an intervention, would not be considered under the healthcare provider perspective conventionally but would be from the perspective of the individual patient as well as under a comprehensive societal perspective. Furthermore, the opportunity cost of resources donated by global health stakeholders/donors (such as donated drugs or vaccines) could be valued based on the price of those goods in the recipient country if the healthcare provider perspective was used compared to the cost it was procured by the donor or an estimated social opportunity cost if the societal perspective was used. The difference between the financial and economic cost of an intervention and what adjustments to market prices are needed will be influenced by the chosen perspective. Importantly even if the healthcare provider perspective is used, economic costs that fall under that perspective should still be considered within economic evaluations (as opposed to only financial costs)	
A related concept to the perspective that is important when considering opportunity costs is the timeframe of the analysis. The timeframe can influence if a resource has an opportunity cost and how economic costs should be valued. For example, it could be argued that in the short term, there are no alternative uses of healthcare facilities, and therefore the opportunity cost for the use of the building space to the healthcare provider or healthcare sector is zero. However, in the longer term, there is potential for alternative uses (such as use in other public activities, or sale to the private sector), and therefore these resources do have an opportunity cost [10]. In addition, sunk costs are costs that have already been incurred and that cannot be retrieved [7]. These should generally be ignored when considering economic costs because they will remain the same regardless of the outcome of a decision and what resources have been used in the past is not a determinant of the optimum decision of how to allocate resources moving forward [1]. However, it is important to note that there can be ongoing opportunity costs related to previously purchased resources (such as the use of building space or vehicles) if they could be used for other services within the timeframe of the study	

**Fig. 1:**
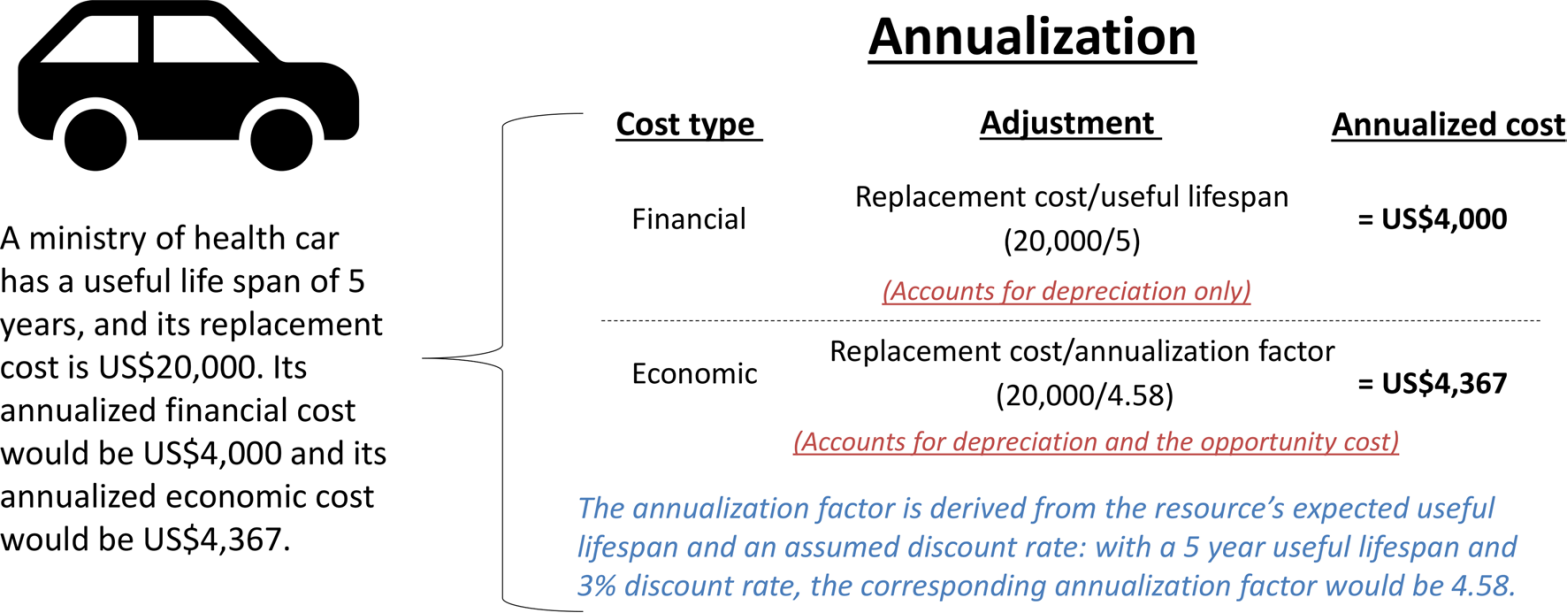
The difference between the annualized financial and economic cost of a capital resource. As capital resources (such as vehicles) are bought in 1 year but used over several years, their cost needs to be spread over their useful life. This adjustment is known as annualization and it has two potential components; depreciation (the reduction in the value of the asset over time due to wear and tear) and the opportunity cost associated with tying up the funds in purchasing the capital item (as there is a lost opportunity to generate gains from investing that capital). When calculating financial costs the annualization calculation only captures depreciation, by dividing its replacement cost by its useful lifespan. In contrast, when calculating economic costs, the annualization calculation also aims to capture the opportunity cost. This is done by dividing the replacement cost by an annualization factor, which is based on the resource’s expected lifespan and an assumed discount or interest rate. Because the annualization factor is a smaller number than the corresponding resource’s expected lifespan (here 4.58 vs. 5), the annualized economic cost will be higher than the annualized financial cost. See Walker et al. [11] for further details

Non-economists are most familiar with the financial (accounting) costs; these represent the actual financial outlays for the goods, resources and services that are purchased. In this context, the financial cost of an intervention represents the amount of money that was paid for the resources being used and they are typically based on expenditure data. However, a difference between financial costs and expenditure data is that financial costs also capture the depreciation in value of capital resources over time (these are the inputs that can be used for more than 1 year, such as equipment and vehicles) [7] (Fig. 1).

In contrast, economic costs represent the full value of the resources utilized in providing an intervention [7]. Crucially, they are intended to capture the resources’ opportunity cost and they are based on the value of the resources’ next-best alternative use that has been forgone due to them being utilised and not simply the monetary amount paid for them. Consequently, with economic costs, all relevant resources consumed by an intervention should be valued, not just those constituting a budgetary line or expenditure. This is a broader conceptualization of a resource’s value than its financial cost and this framework recognizes both that resources can have a value that may not be fully captured by the price that has been paid for it and that by using a resource it makes it unavailable for productive use elsewhere [12]. Table 1 contains a case study illustrating the difference between financial and economic costs for some example resources associated with COVID-19 vaccination campaign in Kenya [13].

**Table 1:** A case study of COVID-19 vaccination at a health clinic in Kenya illustrating the potential differences between financial and economic costs

Resource example	Financial costs	Economic cost
Healthcare personnel	There would be no financial costs related to using the existing healthcare personnel associated with COVID-19 vaccine delivery as they are already receiving a salary	There would be an economic cost associated with having the existing healthcare personnel spend time on COVID-19 vaccine delivery—as they could have used their time on another healthcare program/activity or have more breaks/leisure time
Use of physical space at the health clinic (such as beds or booths used for the vaccine administration)	There would be no financial cost to the COVID-19 vaccination program associated with using the space at the health clinic	There would be an economic cost associated with the time the space is used at the health clinic for COVID-19 vaccine delivery—as the space could have been used for another healthcare program/activities
A subsidised vaccine	The financial cost associated with the COVID-19 vaccines would be valued based on the negotiated price that the government paid for them	The economic cost associated with the COVID-19 vaccines would be based on their market price in the country setting (i.e. Kenya)

A case study illustrating the difference between financial and economic costs for different resources associated with COVID-19 vaccination in Kenya (based on Orangi et al. [13]) using the health system perspective and a 1 year time horizon. Note that this is just an illustration of the principles and not a full list of the costs

Accounting for these hidden costs associated with alternative uses of resources can be more difficult than the more visible financial costs but is important when making resource allocation decisions surrounding healthcare policies. Ultimately, the monetary values given to these economic costs will depend on the research question, context, adopted perspective, and the timeframe being considered (these issues are outlined further in Box 1). Due to this, it can be necessary to use the financial costs incurred for some resources to approximate the economic costs of that resource in its alternative use (particularly under the healthcare provider perspective). However, it is also possible that a resource’s observed financial cost can be lower than its economic cost (such as due to subsidies, discounts or donations) [5] and vice versa when prices are distorted/inflated due to political/economic factors (i.e. taxes/import tariffs) or profit margins in case of services within the private sector [5]. For example, under the societal perspective, the market prices of patented drugs can be much higher than their social opportunity costs (which would only reflect short-run manufacturing and distribution costs) [14, 15].

It is important to note that financial and economic costs are different ways of thinking about and quantifying costs, and not different elements of the overall cost of an intervention (summarized in Table 2).

**Table 2:** Summary of the difference between financial costs versus economic costs (adapted from [16])

	Financial cost	Economic cost
Description	Represent the actual financial outlays for the goods, resources and services that are purchased	Represent the full value of the resources utilized in providing an intervention. Crucially, they are intended to capture the resources’ opportunity cost and they are based on the value of the resources’ next-best alternative use that has been forgone due to them being utilised and not simply the monetary amount paid for them
Costs included	Inputs purchased and the depreciation in value of capital resources over time	All of the resources (that fall under the perspective) not just those constituting a budgetary line or expenditure
Valuation	Market prices of purchased goods	Can use the market prices as a proxy. In the cases where there is no market price or it is believed that the market price does not accurately reflect the opportunity cost, a “shadow price" (an estimated or adjusted value of a good or service) can be used. Note that this valuation will depend on the context of the study, the chosen perspective, and the objective (Box 1)
Purpose	Financial costs have a prominent and justified role for purposes of budgeting and financial planning of health including within budget impact analysis (i.e. assessing the affordability of the intervention)	Economic costs should be used for analyses aimed at assessing the value for money or efficiency of alternative policy options when informing policy decisions i.e. making choices regarding the allocation of scarce healthcare resources (such as within economic evaluations)

Economic costs are expressed in monetary currencies for convenience or comparability. It is also possible to express the opportunity cost of an intervention in other ways. For instance, the opportunity cost could be expressed in natural units (such as alternative patients forgone), or in terms of the alternative health benefits that have been forgone by using those resources (also referred to as health opportunity costs) [17–20]. For example, the opportunity cost of a malaria programme could either be measured by quantifying the economic cost of the programme in monetary terms or by valuing the health that has been foregone by not using the resources the programme utilised for another intervention (its health opportunity costs).

Within this paper, we have focused on financial and economic costs. However, there are other cost types within health economic analysis (such as average, marginal and incremental costs). Further information related to these other cost types are outlined within the Global Health Cost Consortium reference case [7].

### When and why are economic costs preferable over financial costs in health economic analyses

Financial and economic costs are fundamentally different and have different purposes. The type of cost to use will depend on the objective of the study [7]. Financial costs have a prominent and justified role for purposes of budgeting and financial planning of health services because they are paid for by actual financial outlays from a defined budget [7, 21]. For example, financial costs are more appropriate to use if a Ministry of Health was developing a fiscal plan for a clearly-defined budget to implement a particular intervention (such as a vaccination campaign) that has been decided already to be implemented, including within budget impact analysis [22].

In contrast, economic costs should be used for analyses aimed at assessing the value for money or efficiency of alternative policy options when informing policy decisions i.e. making choices regarding the allocation of scarce healthcare resources before the implementation decision is being made [5, 7, 23]. For example, economic costs should be used within economic evaluations of healthcare interventions (such as cost-effectiveness analysis or cost–benefit analysis). This is particularly relevant when using societal or healthcare system perspectives but is even needed when using a healthcare provider perspective. Economic costs are important in this context for several reasons (also highlighted in Box 2):

1. *Informing the optimum allocation of the limited and competing resources available for healthcare*: The resources and funding available for healthcare are explicitly limited. Consequently, to decide whether the additional benefits generated by a health intervention justify its additional costs depends on the value of what is given up as a consequence (the opportunity costs) [15]. The economic cost of an intervention represents the full value of the resources being used and accounts for the fact they could have been used for something else (their opportunity cost). They therefore support the use of economic evaluations to assist with informing the optimum allocation of the finite resources available for public health. For example, giving additional tasks to current full-time healthcare workers will not incur any additional financial costs (assuming they do not need to work overtime for the additional tasks), but their time still has an opportunity cost in terms of the other duties that were not performed (or poorly performed).
2. *Sustainability*: By excluding resources which were not purchased (such as free use of building space, free use of vehicles, or voluntary labour), financial costs can give a misleading indication of the sustainability of an intervention. However, the cost and cost-effectiveness could change significantly if these resources were no longer freely available. Economic costs provide a better indication of the sustainability of the costs of interventions through their link with what the next-best use would have been. In this context, it is important to note in practice, health technology assessment is not usually performed repetitively on a single intervention to inform short term decisions. Hence, it can be important to consider longer timeframes in this context, where the donated or subsidised goods may not be available—even under a payer/healthcare provider perspective.
3. *Replicability*: Financial costs can also give a misleading indication of the replicability of an intervention, i.e. the financial cost of an intervention in a particular setting does not necessarily accurately reflect the cost to replicate the intervention in a similar setting (as the amount of donated resources may be different). Economic costs provide a more comprehensive starting point to extrapolate costs across settings. This is particularly important in the context of LMIC settings, as cost data and economic evaluations are often needed to be generalised across multiple countries.

**Box 2:** The unpaid time of volunteers

Community volunteers are being used within a number of other healthcare interventions: including mass drug administration, vitamin A supplementation, supporting HIV and tuberculosis patients, and community case management of childhood illnesses [24–30]. Voluntary labour is, by definition, free from wages to the health care provider. However, the economic value of the volunteers’ unpaid time is still important to account for, as the time they lose is a genuine economic resource that could have been used on other valuable activities (i.e. they gave up an alternative use of their time—such as paid work or leisure). There are also practical reasons why the economic costs related to volunteers’ time should be considered when evaluating different policy decisions and resource allocation. For example:
∙ Relying on volunteers for such a growing range of roles and interventions could become unsustainable. Potentially, volunteers who in the past worked for free would start expecting to be paid. Thus, ignoring the economic value associated with their time/contribution is important for accounting for the sustainability of the costs of interventions
∙ Community health volunteers are not established in every setting. Therefore, in some cases, more formal healthcare workers will be needed to perform the same tasks. Consequently, accounting for the value of the community health volunteers' unpaid contribution is an important consideration when generalizing cost data to other settings
Consequently, when performing an economic evaluation on an intervention that involves community volunteers, it is important to consider their economic cost, even when using the healthcare provider’s perspective. Despite their importance, the economic costs relating to volunteers' unpaid time are often overlooked or estimated inconsistently. This can give a misleading indication of the sustainability and replicability surrounding the costs of the interventions using community volunteers, potentially leading to inefficient policy decisions
The economic costs can be significant. For example, the economic costs related to the unpaid time contributed by community health volunteers' to mass drug administration for neglected tropical disease control have be found to be notable, with the averages of the different studies varying between US$0.05 and $0.16 per treatment [31]. For comparison a benchmark of US0.50 per treatment is commonly used for the delivery costs for such programs [32], highlighting the significance of these non-financial costs
It should be noted that at times, community health volunteers are given generous per diems and/or incentives, making the distinction between their time being paid or unpaid more difficult. In addition, some economists have argued that volunteers may be willing to supply labour for free since they perceive the benefits of volunteering to outweigh the opportunity costs associated with their time (i.e. it is of benefit to them and not a “cost”) [33]. This highlights the complexities in estimating opportunity costs

Ultimately, the choice of cost type and how resources are valued will depend on what the objective of the study is, what is being optimised and from what perspective and over what timeframe (see Box 1)—i.e. the funds of a specific disease control programme, the spending of a healthcare budget to promote health or gains in social welfare.

### How economic costs are calculated

There are three steps involved in estimating cost values: (1) identifying what resources are used under the studies perspective and timeframe, (2) measuring how much of each resource is used, and (3) placing a value on each resource [4]. In terms of the first and second steps, when estimating economic costs, all the resource items that are involved in the delivery of the health intervention (under the study’s perspective) that are expected to change when the intervention is introduced should be costed and measured, including donated inputs [34]. It is recommended that even resources that were previously purchased (such as buildings and equipment) and resources not currently used to their full capacity should be considered as incurring opportunity costs, if they can be used for other activities/services within the considered timeframe [7] (Box 1).

The complexity surrounding economic costs arises with the third costing step—placing a monetary value on each resource [35]. In theory, when the equilibrium price at which a resource is sold represents its competitive market value, it can be assumed to reflect its opportunity cost: as the market price (the amount of money for what an asset can be sold in a market) represents the resource’s value within its next best alternative use. However, in reality, perfectly competitive markets are rare in healthcare due to the imperfections in healthcare markets (such as taxes, subsidies, price controls and trade barriers). Consequently, unadjusted market prices may not always reflect the theoretical true value of a resource within its next best alternative use [15, 35, 36]. The difference between market prices and value is particularly important for “fees for services” payments which may not be based on actual resource use. For example, the opportunity cost of a hospital bed day is not necessarily adequately represented by what the hospital charges [37] and physician fees may not accurately reflect the relative skill level and time required for different procedures [15].

In practice, the pragmatic approach to costing healthcare interventions is typically to use existing market prices as an approximation to cost each individual input unless there is some particular reason(s) to do otherwise (such as if a resource is being subsidized or donated) [15]. This provides a relatively straightforward and practical approach for estimating economic costs [15, 35] (see examples in Table 1). Within this approach there are three key potential differences in the valuation of economic costs compared to financial costs:

1. The first is placing an economic value on the relevant resources for which no financial costs were incurred (such as donated items including the free use of building space or use of volunteers). This can be based on what the corresponding market price would have been for these resources. However, for resources that do not have a corresponding market price, an estimated “shadow price" (an estimated or adjusted value of a good or service [7]), can be needed to approximate their opportunity cost. For example, there is often no market price for volunteer work or for the time spent by informal caregivers, so an estimated shadow price is needed to value these resources when estimating economic costs. What resources need to be valued will still depend on the perspective (for example any of the costs incurred by patients/caregivers would not be included under the healthcare provider/payer perspective). However, even a under healthcare provider/payer perspective relevant donated resources may be included/valued and can be important drivers in the cost of interventions. This is outlined further using the example of community volunteers in Box 2.
2. The second is the use of an adjusted shadow price for the relevant resources which had a financial cost, but it is believed that the market price does not accurately reflect the opportunity cost. This could include using a shadow price to adjust the price that was paid for a drug or vaccine. Drummond et al. [15] recommended that market prices should only be adjusted when the analyst is convinced that (1) to leave prices unadjusted would introduce substantial biases into the study and (2) there is a clear and objective way of making the adjustments. Whether adjustments to market prices are needed will depend on the perspective of the study. For example, if the healthcare provider/payer perspective was being used, it might be appropriate to use the market prices the provider paid for a drug or vaccine. However, if a societal perspective was being used, these prices may need to be adjusted to reflect their social opportunity costs.
3. The third is related to the valuation of capital resources (resources that have a useful life of over 1 year—such as buildings, vehicles or medical equipment). As these resources are bought in 1 year but used over several years, their cost needs to be spread over their useful life. This adjustment is known as annualization and is different for financial and economic costs (illustrated in Fig. 1). This is because the annualized financial cost only captures depreciation (the reduction in the value of the asset over time due to wear and tear). In contrast, the annualized economic cost also aims to capture the opportunity cost associated with tying up the funds in purchasing the capital item (as there is a lost opportunity to generate gains from investing the money). Due to this the annualized economic cost will be higher than the corresponding annualized financial cost—even from a healthcare provider/payer perspective.

The key point is that when evaluating the economic cost of healthcare interventions is that it is important to not only consider the resources associated with expenditures and that the values of resources may not be adequately reflected by their market prices. What adjustments are needed and the difference between the financial and economic costs will depend on the context of the study (including the perspective taken) and should be clearly reported/justified.

As previously mentioned, the economic cost for a resource can be lower, the same or higher than the corresponding financial cost. At an intervention level, the total economic cost is generally higher than its total financial cost. For example, it was estimated that the total financial cost per person vaccinated with two doses against COVID-19 in Kenya (at a 100% coverage level) was US$16.47, whereas the total economic cost per person was US$24.68 [13]. However, this will not necessarily always be the case and will depend on the context of the study and what adjustments are made.

Further guidelines on how to estimate economic costs for different types of healthcare resources are available; For example, UNAIDS Costing Guidelines for HIV Prevention Strategies [12], Hutton and Baltussen [10], and the WHO’s guide to cost-effectiveness analysis [5]. In addition, more advanced methods can be used to estimate opportunity costs, that are less reliant on market prices [37]. In some situations, the health opportunity cost associated with additional health spending (such as [19]) can be used as a shadow price of the economic cost of an intervention. For example, Sandmann et al*.* [37] calculated the opportunity cost of a hospital bed day by monetising the health forgone from the second-best alternative patient that was unable to use it. This more advanced methodology for estimating the economic costs associated with specific resources is harder to apply in LMIC settings—where there is likely be multiple diseases specific donors instead of a single healthcare provider with a fixed budget, and where the second-best alternative patient may be more challenging to identify. That said it is important to note that health opportunity costs are being used to estimate country specific cost-effectiveness thresholds for LMIC settings [17, 38, 39].

### Issues to be aware of when interpreting economic costs

The following are issues that are important to consider when either estimating or interpreting economic costs.

A source of confusion surrounding economic costs is that there are many subtly different definitions and applications for estimating them within health economics [1, 37]. Ultimately, the different definitions often have similar or even the same meaning, but they cover specific aspects or situations that are not always generalisable.

There can be variation across different studies regarding what costs items are included as financial versus economic costs and how they are valued. This is at least partly driven by differences in the perspective of the analysis and the context of the intervention (which can influence which resources are donated). This variation in how cost items are classified and how they are valued will influence the difference between financial versus economic cost estimates of interventions. It is important to consider this when comparing different studies.

It can be difficult to decide what is an appropriate value to assign the opportunity cost for a particular resource, where there are different prices paid by different purchasers. For example, in the case of development assistance to LMICs, depending on the perspective the opportunity cost of resources donated by global health stakeholders/donors could be based on the price to purchase those goods in the recipient country or the cost it was procured by the donor. The differences between these values will reflect market differences (such as trade barriers keeping local prices high) or inefficient procurement mechanisms (such as not buying the cheapest available product). Furthermore, under the societal perspective, the opportunity cost of using a donated patented drug can depend on if a generic substitute exists (with the same level of effectiveness) and is available in that setting or not. The guiding consideration in this context should be the value nearest to the value of the good in a local perfectly competitive market. Studies need to report these types of assumptions in greater detail, and in some cases explore them within the sensitivity analysis (which tests the robustness of the conclusions by repeating the comparison between inputs and consequences while varying the assumptions used)—as they can have a significant impact on the estimated total cost.

It is important to note, that the use of market prices is a pragmatic approach to estimating economic cost. However, the shadow prices being used within economic cost calculations may not always be a good approximation of opportunity costs (the value of the next best alternative that has been forgone)—particularly under the the societal perspective. For example, the economic cost associated with the time of skilled clinical labour, is typically estimated using a shadow price based on their prevailing market wages (their gross salary and fringe benefits) [5]. However, the opportunity cost associated with the time of skilled clinical labour could also be determined based on the value of the health that is displaced because they are unable to see another patient. This could be particularly notable when skilled clinical labour is scarce. For example, the opportunity cost associated with skilled clinical labour during the COVID-19 pandemic would be significant in terms of lost health, the value of which will not be fully captured by their market wages. This highlights the importance of also considering health opportunity costs.

## Conclusion

Costs are a vital component for economic analysis informing health policy decisions. However, there are different types of costs, and the correct type to use will depend on the type of study and the objective.

Financial and economic costs have different purposes within health economics studies. Financial costs are needed for the purposes of budgeting and planning of health services, as well as understanding the affordability of a new intervention (such as within budget impact analysis). In contrast, economic costs are needed when assessing the value for money of alternative policy options for informing policy decisions i.e. making choices regarding the allocation of scarce healthcare resources. It is important to note that financial and economic costs are different ways of thinking about and quantifying costs, and not different elements of the overall cost of an intervention. However, financial costs are sometimes used as a proxy for opportunity costs when quantifying economic costs.

When evaluating the economic cost of healthcare intervention it is important to not only consider resources that are associated with expenditures, and that the values of resources may not be adequately reflected in market prices. What adjustments are needed and the difference between the financial and economic costs will depend on the context of the study, the chosen perspective, and the objective. In the future studies should more clearly define the methods they used to calculate economic costs. Finally, it is also important to note that the shadow prices based on market prices used within economic cost calculations may not always be a good approximation of opportunity costs—particularly under the societal perspective.

## Data Availability

No datasets were generated or analysed within this study.
